# Distinct Plasma Immune Profile in ALS Implicates sTNFR-II in pAMPK/Leptin Homeostasis

**DOI:** 10.3390/ijms24065065

**Published:** 2023-03-07

**Authors:** Vincent Picher-Martel, Hejer Boutej, Alexandre Vézina, Pierre Cordeau, Hannah Kaneb, Jean-Pierre Julien, Angela Genge, Nicolas Dupré, Jasna Kriz

**Affiliations:** 1 CERVO Brain Research Centre, Department of Psychiatry and Neuroscience, Faculty of Medicine, Université Laval, Québec City, QC G1J 2G3, Canada; 2 CHU de Québec, Department of Medicine, Université Laval, Québec City, QC G1J 1Z4, Canada; 3 Montreal Neurological Institute and Hospital, McGill University, Montreal, QC H3A 2B4, Canada

**Keywords:** plasma, sALS, fast progressors, leptin, sTNF-RII, AMPK, adipocytes

## Abstract

Amyotrophic lateral sclerosis (ALS) is a clinically highly heterogeneous disease with a survival rate ranging from months to decades. Evidence suggests that a systemic deregulation of immune response may play a role and affect disease progression. Here, we measured 62 different immune/metabolic mediators in plasma of sporadic ALS (sALS) patients. We show that, at the protein level, the majority of immune mediators including a metabolic sensor, leptin, were significantly decreased in the plasma of sALS patients and in two animal models of the disease. Next, we found that a subset of patients with rapidly progressing ALS develop a distinct plasma assess immune–metabolic molecular signature characterized by a differential increase in soluble tumor necrosis factor receptor II (sTNF-RII) and chemokine (C-C motif) ligand 16 (CCL16) and further decrease in the levels of leptin, mostly dysregulated in male patients. Consistent with in vivo findings, exposure of human adipocytes to sALS plasma and/or sTNF-RII alone, induced a significant deregulation in leptin production/homeostasis and was associated with a robust increase in AMP-activated protein kinase (AMPK) phosphorylation. Conversely, treatment with an AMPK inhibitor restored leptin production in human adipocytes. Together, this study provides evidence of a distinct plasma immune profile in sALS which affects adipocyte function and leptin signaling. Furthermore, our results suggest that targeting the sTNF-RII/AMPK/leptin pathway in adipocytes may help restore assess immune–metabolic homeostasis in ALS.

## 1. Introduction

Amyotrophic lateral sclerosis (ALS) is an adult onset neurodegenerative disease characterized by a progressive loss of upper and lower motor neurons [1,2]. Most ALS cases are classified as sporadic (sALS ± 90%), while the remaining ±10% are familial (fALS) [3]. Clinical presentations and evolution of the disease are highly heterogeneous between patients [4], even between those carrying the same genetic profile [5]. Although ALS is invariably lethal, approximately 20% of the patients survive >5 years and 10% may survive >10 years after the onset of the first symptoms [6]. On the other side of the spectrum, it has been observed that 10–20% of patients develop a rapidly progressing disease leading to death in less than a year following initial diagnosis [7]. The underlying cause for this high variability in the clinical course of disease remains unclear. The combination of genetic and/or environmental modifying factors may influence the rate of disease progression [8,9]. For instance, juvenile onset or younger age at diagnosis, upper motor neuron-predominant and some form of hereditary ALS are associated with longer survival [6,10,11], while, bulbar onset, low forced vital capacity (FVC < 80%) and lower revised ALS functional Rating Scale (ALSFRS-R) at the first visit are associated with shorter survival [11]. At present, growing evidence suggests that a chronic dysregulation of immunity may significantly affect the course of disease as well as metabolic homeostasis in ALS. It is noteworthy that, one of the conditions associated with a faster rate of functional decline and shorter survival is development of a hypermetabolic state in ALS [12,13].

In the current study, we took advantage of an unbiased multiplex metabolic/immune array approach and measured, at the protein level, sixty-two different cytokines/chemokines/adipokines in the plasma samples collected from sporadic ALS patients (sALS). The proof-of concept sample analyses revealed that some of the key immune mediators were either downregulated and/or not regulated. To our surprise, the metabolic sensor leptin was identified as the most consistently deregulated protein in the plasma of sALS samples. Of note, the observed deregulation was more pronounced in male patients. Similar observations were made in different mouse models of ALS, notably the SOD1^G93A^ mice and the double transgenic TDP-43^G348C^; UBQLN2^P497H^ mice. Normally implicated in regulation of food intake, growing evidence suggests a role for leptin in inflammation, neuroprotection, as well as learning and cognitive functions [14,15,16,17,18]. Prior reports have suggested a potential role of leptin in ALS pathogenesis, as its levels are inversely associated with the risk of developing disease [19,20]. Furthermore, studies in the TDP-43^A315T^ mouse model revealed a reduction in leptin levels at the end-stage of disease [21], while treatment with the recombinant protein improved motor performance and delayed weight loss in this mouse line [22].

Next, we identified a unique immune–metabolic plasma molecular signature associated with fast progressing ALS, characterized by: (i) elevated levels of soluble TNF receptor II (sTNF-RII) and C-C motif chemokine ligand 16 (CCL16), (ii) further decrease in plasma levels of leptin when compared to slow sALS. In a search for underlying mechanisms, a series of in vitro experiments revealed that human adipocytes exposed to plasma of fast sALS and/or recombinant sTNF-RII reproduced a similar immune–metabolic response resulting in a marked downregulation of leptin secretion. The observed reduction in leptin correlated with an increase in AMP-activated protein kinase (AMPK) phosphorylation levels in adipocytes, a principal sensor implicated in leptin production. Conversely, treatment with AMPK inhibitors restored leptin production in human adipocytes. Together, this study provides in vitro and in vivo evidence of a unique immune/metabolic profile in plasma of sALS, and in particular fast progressing ALS patients, which alters adipocyte function and leptin homeostasis.

## 2. Results

Growing evidence suggests that a chronic deregulation of immunity may represent one of the key elements in the pathobiology of neurodegenerative disorders such as ALS and it may contribute to the observed heterogeneity in the rate of disease progression and/or regulation of metabolic homeostasis [23,24,25]. To assess immune–metabolic profiles in ALS, we recruited 51 sALS patients and 38 age-matched controls. Patients were eligible for inclusion if they had a definite or probable diagnosis of ALS based on El-Escorial criteria, were aged at least 25 years, and had no familial history or genetic cause of ALS. Demographics were very similar between patients and controls for weight, body mass index (BMI) and age (Table 1).

As presented in Table 2, the patients were categorized as slow and fast progressors based on difference in ALSFRS-R score between two-time points. Patients were classified as fast progressors if they lost more than 4 points/12 weeks in ALSFRS-R [16]. Using these criteria, 11 patients were classified as fast progressors with a mean loss of 7.884 points/12 weeks as compared to a mean loss of 1.683 points/12 weeks in slow progressors (*p* < 0.0001).

### 2.1. Reduced Levels of Immune Mediators and the Metabolic Sensor Leptin in Plasma of Sporadic ALS Patients

**Figure 1:**
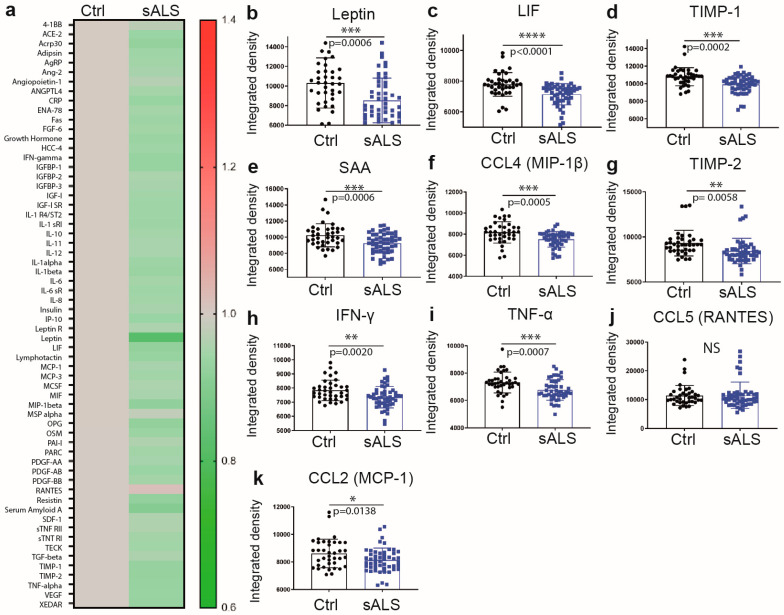
Cytokines/adipokines/chemokines profile in sALS patients. (**a**) Heat map illustrating changes in cytokines/adipokines and chemokines levels (ratio) in sALS patients (51) as compared to healthy controls (38) measured by cytokines array. (**b**–**k**) individual graphs with significant variations. Human Obesity Array is a semi-quantitative method with arbitrary measures (integrated density) (Data are mean ± SEM * *p* ≤ 0.05, ** *p* < 0.01, *** *p* < 0.001, **** *p* < 0.0001, NS, not significant).

To assess the effects of disease on immune and metabolic signaling in sporadic ALS, we took advantage of an unbiased screening array and measured plasma levels of 62 different cytokines and adipokines. To our surprise, quantitative analysis revealed that the protein levels of the majority of immune mediators including different cytokines and chemokines were decreased in plasma of sALS patients as compared to controls (Figure 1a). We detected a significant reduction in levels of leukemia inhibitory factor (LIF, −8.64%, *p* < 0.0001) (Figure 1c), tissue inhibitor of metalloproteinase 1 (TIMP-1, −7.70%, *p* = 0.0002) (Figure 1d), serum amyloid A (SAA, −10.03%, *p* = 0.0006) (Figure 1e), chemokine (c-c motif) ligands 4 (CCL4, −8.57%, *p* = 0.0005) (Figure 1f), TIMP-2 (−9.90%, *p* = 0.0058) (Figure 1g), interferon-gamma (IFN-γ, −6.24%, *p* = 0.0020) (Figure 1h), tumor necrosis factor-alpha (TNF-α, −8.01%, *p* = 0.0007) (Figure 1i), CCL2 (−6.16%, *p* = 0.0138) (Figure 1k) and metabolic sensor leptin (−17.95%, *p* = 0.0006) (Figure 1b). This is in accordance with previous reports suggesting lower leptin levels in ALS patients [19]. Of note, correlation has been found between leptin levels and BMI (R^2^ = 0.08669, *p* = 0.0422) (Figure S1A). Importantly, leptin levels did not change in patients treated with riluzole (Figure S1D).

### 2.2. Differential Increase in Plasma Levels of sTNF-RII and CCL16 Is Associated with Fast Progressing Disease

**Figure 2:**
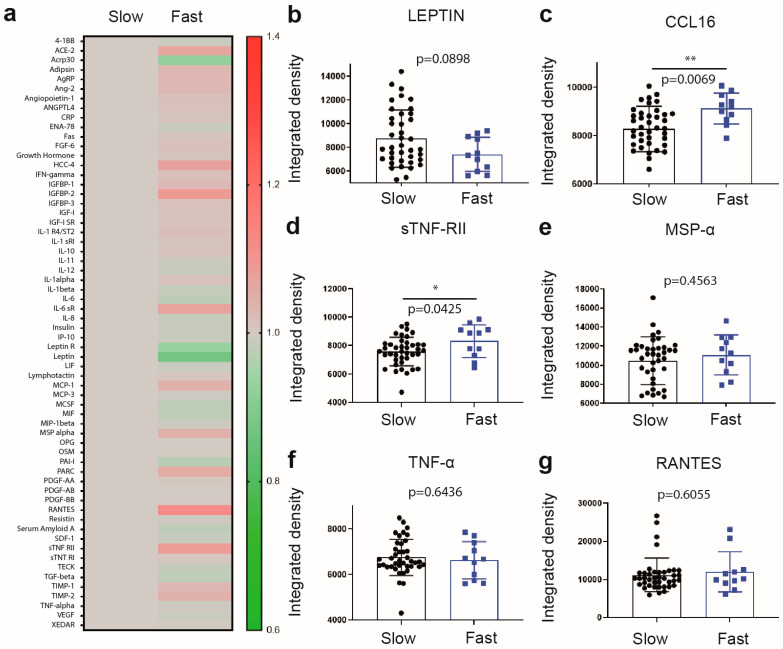
Cytokines/adipokines/chemokines profile in fast progressing ALS. (**a**) Heat map illustrating cytokines changes (ratio) in fast progressing ALS patients (11) as compared to slow progressing ALS patients (40) measured by cytokines array. (**b**–**g**) individual graph of cytokines with significant variations. Human Obesity Array is a semi-quantitative method with arbitrary measures (integrated density) (Data are mean ± SEM * *p* ≤ 0.05, ** *p* < 0.01).

Next, we searched for potential differences in immune/metabolic profiles between distinct clinical subgroups of sALS patients classified as slow (<4 points/12 weeks lost) and fast progressors (>4 points/12 weeks lost), slow and fast ALS, respectively. Comparative analyses revealed a significant difference in the plasma levels of distinct immune mediators. Levels of CCL16 (+9.28%, *p* = 0.0069) (Figure 2c) and soluble TNF receptor 2 (sTNF-RII, +8.78%, *p* = 0.0425) (Figure 2d) were significantly increased in the plasma of fast ALS when compared to slow ALS and/or controls. In addition, we observed a marked tendency for lower plasma levels of leptin in fast ALS (Figure 2b). These findings were further confirmed by ELISA assay.

**Figure 3:**
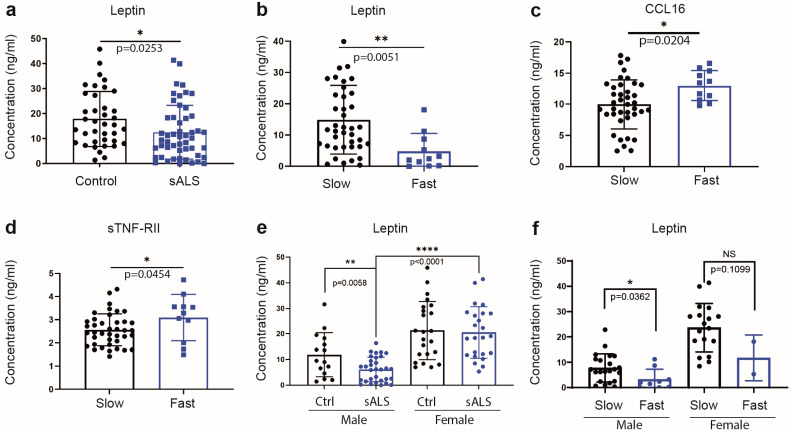
Plasma levels of leptin, CCL16 and sTNF-RII measured by ELISA. (**a**) concentration (ng/mL) of leptin measured by ELISA in healthy controls (38) and sALS (51). (**b**,**d**) Concentration (ng/mL) of leptin (**b**), CCL16 (**c**) and sTNF-RII (**d**) measured by ELISA in slow (40) and fast (11) progressing ALS patients. (**e**,**f**) Concentration (ng/mL) of leptin in sALS and controls sub-grouped by sex (**e**) and by sex and rate of progression (**f**) (Data are mean ± SEM * *p* ≤ 0.05, ** *p* < 0.01, **** *p* < 0.0001, NS, not significant).

As shown in Figure 3a, quantitative analyses revealed a significant increase in reduction in levels of leptin in sALS patients (12.53 ng/mL) as compared to age-matched controls (17.83 ng/mL) (*p* = 0.0253) (Figure 3a). Furthermore, leptin levels were significantly reduced in plasma of fast (4.787 ng/mL) as compared to slow ALS (14.91 ng/mL) (*p* = 0.0051) (Figure 3b). Next, in accordance with the results obtained in protein array analyses, CCL16 and sTNF-RII levels were significantly increased in plasma of fast ALS (13.01 ng/mL) as compared to slow ALS (9.994 ng/mL) (*p* = 0.0204) (Figure 3c) and (3.093 ng/mL vs. 2.563 ng/mL) (*p* = 0.0464) (Figure 3d), respectively. No correlation was found between CCL16 or sTNF-RII levels and BMI or riluzole treatment (Figure S1B,C,E,F). Further analyses revealed significant sex-specific differences in the plasma concentration of leptin in sALS. The levels of leptin in plasma of female sALS patients were highly variable and not significantly changed when compared to age-matched female controls. In contrast, plasma levels of leptin where significantly decreased in all male sALS patients as compared to age-matched male controls (*p* = 0.0058), age-matched female controls (*p* < 0.0001) and female sALS patients (*p* < 0.0001) (Figure 3e). Furthermore, a comparative analysis of leptin levels in the plasma of fast vs. slow ALS male patients revealed a significantly lower concentration of leptin in male patients suffering from the fast progressing disease (Figure 3f). Of note, although we observed an important tendency towards lower plasma leptin levels in fast ALS female patients, the number of fast progressing female sALS patients was too low to achieve statistical significance. Importantly, we observed no sex-specific differences in plasma levels of CCL16 and sTNF-RII. 

### 2.3. Early Deregulation of Leptin in Plasma of SOD1^G93A^ Mice

**Figure 4:**
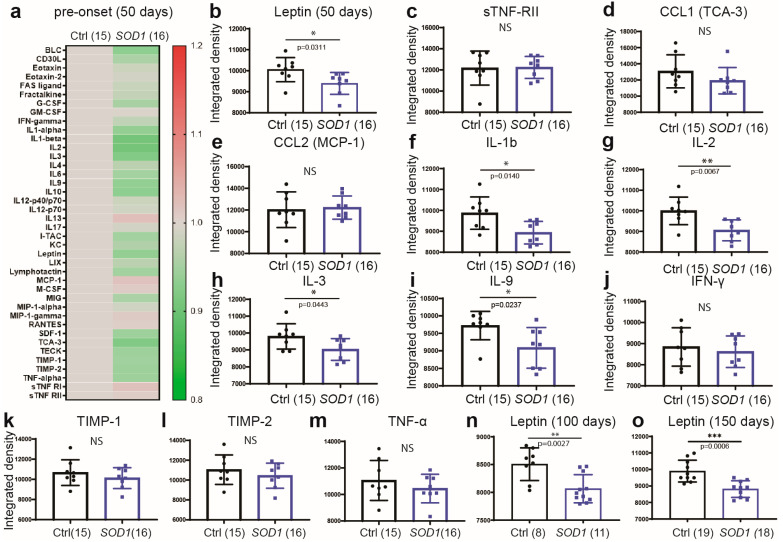
Plasma cytokine profile in pre-onset SOD1^G93A^ mice. (**a**) Heat map illustrating cytokine changes (ratio) in SOD1^G93A^ mice as compared to non-transgenic age-matched mice. The total number of mice is in parentheses (ctrl female = 8, ctrl male = 7, SOD1 female = 9, SOD1 male = 7). Significant changes were detected in leptin (**b**), Il-1b (**f**), IL-2 (**g**), IL-3 (**h**) and Il-9 (**i**). Leptin levels in plasma of mild symptomatic (100 days) (**n**) and advanced stage (150 days) (**o**) SOD1^G93A^ mice. (**c**–**e**,**j**–**m**), the observed changes were not significant. (Data are mean ± SEM * *p* ≤ 0.05, ** *p* < 0.01, *** *p* < 0.001, NS, not significant).

We next investigated whether changes in metabolic and immune profiles observed in human sporadic disease were also replicated in the model of inherited disease, the SOD1^G93A^ mouse [5,26]. By using a mouse model with a predictable disease onset at approximately 100 days of age, we investigated whether certain changes in immune and/or metabolic profile, such as deregulation of leptin occur early in disease, i.e., prior to the onset of clinical symptoms. The assessed immune–metabolic profiles were analyzed at three different time points of disease. As revealed in Figure 4a, very early in disease and before the onset of clinical symptoms (pre-onset), the plasma levels of the majority of cytokines/chemokines were either reduced and/or unchanged (Figure 4a). The levels of leptin in plasma of the SOD1^G93A^ mice were significantly decreased as compared to age-matched non-transgenic controls (*p* = 0.0311) (Figure 4b). We also observed a decrease in the plasma levels of IL-1β (*p* = 0.0140), IL-2 (*p* = 0.0067), IL-3 (*p* = 0.0443) and IL-9 (*p* = 0.0237) (Figure 4f–i), while the levels of sTNF-RII, CCL1, CCL2, IFN-γ, TIMP-1, TIMP-2 and TNF-α remained unchanged (Figure 4d,e,j–m). As disease progressed, at the time of the clinical onset of disease (mild stage) as well as in advanced disease, the plasma leptin levels remained significantly reduced in SOD1^G93A^ mice as compared to non-transgenic mice (*p* = 0.0027), Figure 4o, and (*p* = 0.0006), Figure 4n, respectively. See Figures S2 and S3 for other variable changes in cytokine levels associated with disease evolution.

We next analyzed plasma levels of leptin in two additional experimental models of ALS carrying different disease causing mutations, including TDP-43^G348C^ mice and the double transgenic TDP-43^G348C^; UBQLN2^P497H^ mice [27]. Leptin levels were significantly reduced in the double transgenic UBQLN2^P497H^; TDP-43^G348C^ mice at the mild symptomatic stage (8 months of age) (*p* = 0.0311), but not in the single transgenic TDP-43^G348C^ mice (Figure S4A). Of note, the double transgenic mice exhibit motor deficits with associated neuronal loss, whereas single transgenic TDP-43^G348C^ mice develop a frontotemporal dementia-like phenotype characterized by age-dependent cognitive decline and do not exhibit motor neuron death. Finally, we analyzed and compared leptin levels in plasma of male and female ALS mice. Interestingly, in early disease, the plasma levels of leptin were lower in male SOD1^G93A^ mice as compared to female age-matched mice (Figure S4B). This tendency is not observed in more advanced stages of the disease, since both sexes exhibit a significant disease associated decrease in the plasma leptin levels (Figure S4C,D).

### 2.4. Downregulation of Leptin Correlates with Hyperactivation of the AMPK Pathway in Adipose Tissue of the SOD1^G93A^mice

Leptin production in adipocytes is in part controlled by the metabolic sensor AMPK and mammalian target of rapamycin (mTOR) pathways [28]. However, to what extent changes in AMPK activation patterns and phosphorylation at the periphery (i.e., in adipose tissues and/or adipocytes) are associated with a marked reduction in plasma levels of leptin observed in human and mouse disease remains elusive.

**Figure 5:**
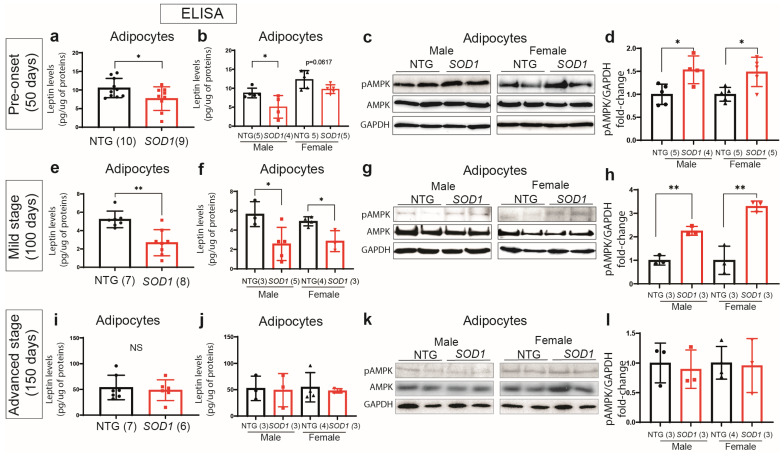
Leptin production by SOD1^G93A^ mice adipocytes. Total proteins from SOD1^G93A^ mice fat tissue were extracted with RIPA buffer as detailed in material and methods. (**a**) Levels of leptin measured by ELISA from adipocytes’ total protein extract in all mice (mixed groups). Concentration of leptin (pg/mL) is normalized with total protein (pg/μg of proteins). Data points represent the mean of two duplicates for each mouse (**b**) Levels of leptin in male and female SOD1^G93A^ mice. (**c**) Immunoblot illustrating levels of phospho-AMPK, AMPK and GAPDH in two females and two males per group. (**d**) Immunoblot quantification of phospho-AMPK vs. GAPDH in SOD1^G93A^ mice as compared to non-transgenic age-matched mice. The same experiments were performed on mild stage (**e**–**h**) and advanced stage (**i**–**l**) mice. No significant change sin leptin levels were observed at advanced stages of disease (NS).The number of mice per group are in parentheses (Data are mean ± SEM * *p* ≤ 0.05, ** *p* < 0.01).

First, we analyzed levels of leptin in adipocyte extracts by ELISA in pre-onset, mild stage, and advanced stage SOD1^G93A^ mice. As shown in Figure 5a,b, early in disease we observed a small but significant decrease in leptin levels in male SOD1^G93A^ adipocytes (Figure 5a,b) and this was peaking at the time of disease onset (Figure 5e,f). To our surprise, in advanced disease, we did not detect a reduction in leptin production by adipocytes (Figure 5i,j), suggesting that it may represent early disease pathogenesis. As further demonstrated in Figure 5g,h, Western blot analysis of adipose tissue homogenates revealed an increase in phosphorylated-AMPK adipocyte extracts in both, male and female mice (Figure 5c,d,h). To our surprise, in advanced disease, we did not detect a reduction in leptin production by adipocytes and observed an overall increase in leptin levels in both non-transgenic and SOD1^G93A^ mice compared to younger mice (Figure 5i,j). The discrepancy between the observed downregulation of plasma leptin in SOD1^G93A^ mice and the similar adipocyte leptin secretion may potentially be explained by the noticeable fat atrophy, which leads to a total reduction in plasma leptin. Residual fat tissue may compensate by increasing its production of leptin. Indeed, a previous report has shown that end-stage TDP-43^A315T^ mice exhibit an increase in adipocyte leptin mRNA levels associated with a downregulation of leptin plasma levels [21]. The observed increase in leptin production in non-transgenic mice may be explained by the significant fat gain in C57Bl/6 at this age. Together these findings suggest that adipocytes’ metabolism, leptin homeostasis and AMPK activation/function may be implicated in early pathogenic mechanisms in ALS.

### 2.5. Production of Leptin in Human Adipocytes Is Regulated by AMPK

**Figure 6:**
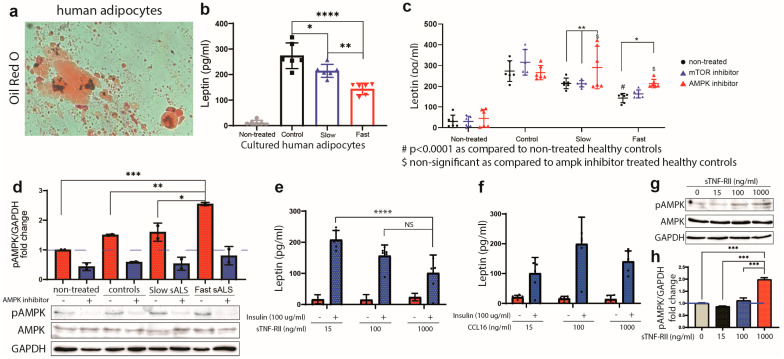
Exposure to plasma from sALS reduces leptin production in human adipocytes. (**a**) Oil red O coloration demonstrating lipid droplet accumulation (red) in mature adipocytes. (**b**) Human adipocytes were treated for 12 h with 1:100 diluted mixed plasma samples from healthy controls (12), slow progressors (12) and fast progressors (6). The supernatant was collected, and leptin levels were measured by ELISA (pg/mL) and normalized with plasma sample levels of leptin. (**c**) Leptin levels (pg/mL) secreted by mature adipocytes after plasma samples treatment. The adipocytes were pretreated with 1 μM of mTOR inhibitor (PP242) or 10 μM of AMPK inhibitor (compound C) for 1h. (**d**) Proteins from treated adipocytes (**c**) were extracted using RIPA buffer. Graph exhibits pAMPK/GAPDH fold change observed by immunoblotting with or without 10 μM AMPK inhibitor pretreatment. (**e**,**f**) Leptin levels (pg/mL) secreted by mature adipocytes after insulin and sTNF-RII treatment (**e**) and insulin and CCL16 treatment (**f**). (**g**) Immunoblotting showing pAMPK levels in adipocytes treated with increasing concentration of sTNF-RII. (**h**) Quantification of immunoblotting in (**g**) showing levels of pAMPK/GAPDH fold change in adipocytes treated with sTNF-RII ((Data are mean ± SEM * *p* ≤ 0.05, ** *p* < 0.01, *** *p* < 0.001, **** *p* < 0.0001) *n* = 3, two-way ANOVA). NS, not significant.

To further investigate the mechanisms involved in (de)-regulation of leptin secretion in ALS, we created a controlled humanized in vitro model-system. As shown in Figure 6a, the human adipocytes were differentiated and matured (contained lipid droplets) as revealed by positive Oil Red O coloration. The adipocytes were then conditioned for 12 h with pooled plasma samples from 12 healthy controls, 12 slow and 6 fast ALS. Importantly, mimicking the findings obtained in human disease in vivo, the adipocytes treated with fast ALS plasma samples had reduced production of leptin as compared to healthy controls and slow ALS (Figure 6b). In addition, the adipocytes exposed to plasma of the fast ALS expressed significantly higher levels of pAMPK as compared to slow progressors and controls (Figure 6d). As AMPK acts as the principal sensor implicated in leptin production, next we tested whether reduction in leptin production/secretion could be abrogated by the inhibition of AMPK. The adipocytes were pretreated for 1 h with an AMPK inhibitor (compound C, 10 μM) or a mTOR inhibitor (PP242, 1 μM) and then exposed for 12 h to different plasma samples. The mTOR inhibitors were used as a negative control since the mTOR pathway would normally be inhibited in the context of AMPK hyperphosphorylation. As shown in Figure 6d, AMPK inhibitors were efficient in reducing AMPK phosphorylation. Further analysis revealed that the pre-treatment with AMPK inhibitor restored leptin levels in human adipocytes conditioned with plasma from slow (*p* < 0.0001) and fast ALS (*p* = 0.0169) (Figure 6c). As expected, the pre-treatment with mTOR inhibitors had no impact on leptin secretion.

### 2.6. sTNF-RII Reduces Leptin Production in Human Adipocytes

Our results so far suggest that the deregulation of immune profile observed in fast ALS, may have an additional impact on leptin production by adipocytes. Given that sTNF-RII and/or CCL16 are differentially increased in plasma of the fast progressing patients, we investigated their impact on leptin production in cultured adipocytes. To maximize leptin production and release, human adipocytes were treated with insulin which activates leptin release via PI3K/AKT activation in humans [29,30]. Next, the cells were exposed to different concentrations of recombinant proteins sTNF-RII and/or CCL16. As shown in Figure 6e, sTNF-RII blocked the insulin-driven production of leptin at 1000 ng/mL while treatment with CCL16 did not have a significant impact on leptin production (Figure 6f). Furthermore, exposure of adipocytes to sTNF-RII significantly increased the levels of pAMPK in a dose-dependent manner (*p* ≤ 0.0002) (Figure 6g,h).

## 3. Discussion

Chronic deregulation of immunity is a hallmark of many neurodegenerative disorders including ALS. Here, we provide in vivo evidence of a chronic deregulation of plasma immune profile and leptin homeostasis in human disease, as well as in an experimental ALS model, notably the SOD1^G93A^ mouse. We report a reduction, at the protein level, of several immune mediators (LIF, TIMP-1, TIMP-2, SAA, MIP-1β, IFN-γ, TNF-α and MCP-1) together with a marked and consistent decrease in the levels of the metabolic sensor leptin. A similar molecular profile was observed in plasma of the SOD1^G93A^ mice. Importantly, both human and mice data suggested a shutdown of peripheral immune response/signaling, and disease evolution associated with a marked deregulation of leptin homeostasis.

To date, the role of immunity in ALS remains controversial. Immune markers have been mainly studied in CSF and much evidence suggests increased levels of several cytokines/chemokines, including IL-2, IL-6, IL-8,IL-10, MCP-1, IL-18 IL-15, MIP-1β, MIP-1α and IFN-γ [31,32,33,34]. However, several conflicting results were obtained following analyses of immune markers in the blood of ALS patients. For example, IFN-γ was found to be increased in some studies [35,36,37], while Lu et al., and Polverino et al., found decreased levels of IFN-γ at the periphery [38,39]. The same discrepancy applies to TNF-α [37,39,40], IL-6 [41,42,43] and others [44]. Furthermore, while cell specific analysis of the immune profiles of peripheral blood monocytes from patients with ALS revealed a marked proinflammatory phenotype at the RNA level [45], some of the observed findings did not correlate with measured proteins, suggesting a certain dissociation of immune profiles at the RNA and proteins levels. The conflicting results in the literature could be in part explained by the variable approaches used for measurement, including individual ELISA, cell population studies, RT-qPCR or multiplex assays of several cytokines [44]. Our study may have had several technical advantages. First, we used an unbiased approach measuring numerous cytokines and metabolic markers at the protein level instead of targeting a unique cytokine. Second, the analysis was always performed in less than 1-week-old samples, reducing the risk of protein degradation and variability in both groups.

Of importance, in patients with fast progressing disease we observed a differential increase in sTNF-RII and CCL16 plasma levels and further decrease in the plasma levels of leptin, i.e., significantly decreased leptin when compared to plasma levels observed in slow progressing ALS. Interestingly, we observed marked sex differences in the plasma levels of leptin in both human patients and SOD1^G93A^ mice. Leptin levels were significantly decreased in the plasma of male, but not in female sALS. It has been established that at any age, leptin levels in females are generally 40% higher than in male [46,47]. At present, the underlying causes of this dimorphism remain unclear, but it is thought to be associated with the fat metabolism associated with reproductive function and steroid levels but doesn’t seem to be associated with body mass index nor total body fat [48]. While it is possible that the observed sex differences may in part explain variability in the plasma leptin levels detected in sALS patients, nevertheless, our data strongly suggests that deregulation of leptin homeostasis remains a pathogenic factor in the subset of sALS patients with fast progressing disease. Indeed, leptin levels were reported to be inversely correlated with disease onset and progression in ALS, suggesting a protective role of leptin in the disease [19].

At present, it is unclear how chronic deregulation of leptin homeostasis in sALS may contribute to disease pathogenesis. Leptin acts on the hypothalamus to regulate energy balance and food intake, but evidence suggests its involvement in some central nervous system pathways. Leptin has been related to neuroprotection after spinal cord injury or stroke [15,49]. It reduces amyloid load and tau phosphorylation in Alzheimer’s disease [50,51,52], as well as dopaminergic cell death in Parkinson’s disease [53], and favors neurogenesis and synaptogenesis [54]. Finally, in accordance with previous work, the results of our study revealed that lower plasma leptin levels may represent a risk factor associated with the faster rate of disease progression [19]. While here we aimed to explore the interactions between immune response and the leptin/pAMPK signaling at the periphery, previous work suggests that leptin may protect against ALS by its direct action on motor neurons and/or its modulation on glial cells’ activity [55], or potentially by its impact on the hypothalamic secretion of different bioactive peptides [56].

The important question here is whether and/or to what extent alterations in the peripheral immunity contribute to the observed deregulation of leptin homeostasis in ALS, in particular, in patients with rapidly progressing disease. Here, it is noteworthy that, in our hands, elevated plasma levels of sTNF-RII were detected in the subset of patients with more rapidly progressing disease. Although sTNFR-II has been previously shown to be elevated in ALS, to date, there are no comprehensive studies analyzing its role on the rate of disease progression [57]. Previous work suggests that under pathological conditions associated with chronic inflammation such as multiple sclerosis, type 2 diabetes and/or cardiovascular disease, TNF-RII is detached from the cell surface through activation of the tumor necrosis factor-alpha converting enzyme (TACE), thus promoting the aberrant immune and noxious response of mononuclear cells [58,59,60,61,62,63]. Furthermore, results from our study strongly suggest that higher plasma levels of sTNFR-II, together with the concurrent hyperactivation of AMPK signaling contribute to reduced production of leptin in ALS-affected adipocytes. Many lines of evidence support this conclusion: (i) cultured human adipocytes released less leptin when conditioned with plasma from fast ALS; (ii) inhibition of AMPK signaling in human adipocytes exposed to plasma from fast ALS restored leptin production; (iii) treatment of human adipocytes with recombinant sTNF-RII induced overexpression of pAMPK and AMPK signaling and blocked leptin production and (iv) the lower levels of leptin correlated with the levels of pAMPK in SOD1^G93A^ mouse adipocytes. While in ALS models, the role of AMPK has not been extensively studied at the periphery, previous work has suggested increased AMPK activity in SOD1 spinal cord culture and a motor neuron cell line [64,65,66]. Indeed, AMPK is hyperphosphorylated in SOD1^G93A^ spinal cord lysates and in *C. elegans* SOD1^G85R^ motor neurons [64,67]. AMPK hyperactivation was also detected in motor neurons of ALS patients [66]. At present, the pharmacological regulation of AMPK has retrieved conflicting results and it remains unclear whether AMPK activation is deleterious or beneficial in ALS. Indeed, activation of AMPK in SOD1^G93A^ accelerated disease onset and progression in female mice [68]. Other data suggested that phospho-AMPK is reduced in mesenchymal stem cells from ALS patients and that AMPK activation may restore neuronal differentiation potency [69]. Taken together, our results illustrate that AMPK may have a distinct pathological profile/function in adipocytes as compared to neurons and other tissues. The specific inhibition of phospho-AMPK in adipocytes may restore leptin production and thus increase levels of leptin, which appear to be related to disease progression.

In summary, we described a unique immune/metabolic profile in ALS patients and SOD1^G93A^ mice. Using an unbiased approach we identified leptin as the most dysregulated assessed immune–metabolic mediator in plasma of sporadic sALS patients, in particular in men with rapidly progressing disease. Next, we showed that exposure to plasma from the fast progressing patients may have a direct impact on human adipocytes’ metabolism and leptin production/secretion via sTNFRII/AMPK signaling. Together, our results suggest that targeting the sTNF-RII/AMPK/leptin pathway in adipocytes may help restore metabolic homeostasis and potentially reduce the rate of decline in ALS patients with rapidly progressing disease.

## 4. Materials and Methods

### 4.1. Recruitment and Samples Preparation

Patients were eligible for inclusion if they had a definite or probable diagnosis of ALS based on El-Escorial criteria, were aged at least 25 years, and had no familial history or genetic cause of ALS. Controls were generally the husband/wife of the patients, when willing to participate. Samples were collected at patients’ homes using EDTA collecting tubes. Samples were centrifuged at 10,000 RPM for 10 min. The supernatant was collected and snap-frozen in liquid nitrogen. This study was conducted according to the guidelines of the Declaration of Helsinki and approved by the Institutional Review Board (or Ethics Committee) of “CHU de Québec” (ethic code: 2021-5742, protocol renewal approved on 27 May 2022).

### 4.2. Human and Mouse Cytokines Array

The Human Obesity Array C1 (RayBiotech, Peachtree Corners, GA, USA, #AAH-ADI-1-8) was performed on human plasma samples, less than one week from sample collection. The array was conducted according to the manufacturer’s protocol. Briefly, the samples were diluted in blocking buffers (1:10). After blocking, membranes were incubated overnight with biotinylated antibody cocktails followed by two hours with HRP-streptavidin. After washing, membranes were developed using high-resolution films. Cytokine intensities were measured using ImageLab Touch Software; Version 2.4.0.03. Each membrane contains six positive internal controls used for data normalization. The RayBiotech analysis tool employs positive controls in one membrane and normalizes to the positive controls in every membrane and therefore, normalizes the cytokine levels, ensuring consistency between every array performed and between both groups. Here, normalization without background suppression was used. In all experiments using human samples we used one membrane per sample. The same protocol was used for comparative analysis of mouse plasma samples, Cytokine Array C3 (RayBiotech, Peachtree Corners, GA, USA, #AAM-INF-1-8).

### 4.3. Experimental Animals

Experiments were performed on wild-type non-transgenic (C57Bl/6), pre-symptomatic, mild and advanced symptomatic SOD1^G93A^, as well as mild symptomatic 8 months of age TDP-43^G348C^ and double transgenic UBQLN2^P497H^; TDP-43^G348C^ mice. Many of the experiments were performed on SOD1^G93A^ mice since this model develops most of the disease characteristics. SOD1^G93A^ mice (B6SJL-TgN (SOD1*G93A)1Gur/j) were acquired from the Jackson Laboratory (Bar Harbor, ME, USA) and genotyped as suggested by Jackson Laboratory protocols. TDP-43^G348C^ and UBQLN2^P497H^; TDP-43^G348C^ transgenic mice were generated and genotyped as described in [27], respectively. TDP-43^G348C^ develops cognitive deficits without motor impairment but double transgenic mice develop both cognitive and motor impairment from 8 months of age. Both males and females were used for experiments. All the experimental procedures were approved by the Laval University Animal Care Ethics Committee and are in accordance with the Guide to the Care and Use of Experimental Animals of the Canadian Council on Animal Care.

### 4.4. Blood and Tissue Collection, Protein Extraction, and Immunoblotting

Mice were sacrificed at 50, 100 or 150 days of age to collect samples for plasma and tissue analysis. Mice were anesthetized via an intraperitoneal injection of ketamine/xylazine (100–10 mg/kg). Blood was removed from the mice by direct sampling from the heart and mice were then slowly perfused with saline. Blood samples were centrifuged at 10,000 RPM for 10 min and supernatant was kept for analysis. Abdominal fat was removed and snap-frozen in liquid nitrogen for protein extraction. Abdominal fat was homogenized in buffer (20 mM tris pH 7.8, 137 mM NaCl, 2.7 mM KCl, 1 mM MgCl_2_, 1% triton X-100, 10% glycerol, 1mM EDTA, 1mM dithiothreitol and 1X proteases and phosphatase inhibitor cocktails). The lysate was sonicated and incubated on ice for 30 min and centrifuged at 13,000 rpm for 30 min and supernatant kept for analysis. The top lipid layer after centrifugation was not collected with the supernatant. Antibodies used for immunoblotting were phospho-AMPKα (Cell signaling, Danvers, MA, USA, # 2531), AMPKα (Cell signaling, Danvers, MA, USA, # 2532) and GAPDH (Santa-Cruz, Dallas, TX, USA, Sc-32233). The immunoblots were developed using the ChemiDoc MP Imaging System (Bio-Rad, Hercules, CA, USA) and the ImageLab Touch Software; Version 2.4.0.03.

### 4.5. Pre-Adipocyte Culture and Treatment

Human primary subcutaneous pre-adipocytes ATCC, (PCS-210-010) were cultured in T75 flasks with fibroblast basal medium containing growth kit-low serum (ATCC, Manassas, VA, USA, # PCS-201-041) (5 ng/mL rh FGFb, 7.5 mM L-glutamine, 50 mg/mL ascorbic acid, 1 mg/mL hydrocortisone/hemisuccinate, 5 mg/mL rh insulin and 2% fetal bovine serum). Maintenance was performed by changing medium every 48 h until cells reached 80% confluence and were ready for sub culturing. Cells were washed using 5 mL D-PBS, trypsinized and split into multiple T75 flasks for amplification. When pre-adipocytes reached 80% confluence, they were trypsinized again and split into 6-well plates with approximately 170,000 cells/well with 2 mL of fibroblast basal medium. After 48 h, we began the initiation phase of the adipocyte differentiation procedure by removing old media and adding 2 mL of adipocyte differentiation initiation medium (ATCC, Manassas, VA, USA, #PCS-500-050) (15 mL adipocyte basal medium (grow kit-low serum and 1 mL AD supplement). A total of 1 mL of media was removed and replaced with 2 mL of fresh adipocyte differentiation initiation medium. From this step forward, adipocytes were never exposed to air to ensure that lipid vesicles did not burst, as suggested by the manufacturer. After 48 h, 2 mL of media was replaced with adipocyte differentiation maintenance medium (ATCC, Manassas, VA, USA, #PCS-500-050) (85 mL basal medium with 5 mL ADM supplement). This step was repeated every 72 h for a total of 15 days from initiation phase until adipocytes reached full maturity. To examine the impact of sALS plasma on leptin production, mature human adipocytes were treated with 1:100 diluted pooled plasma (in differentiation maintenance medium) from either 12 healthy controls, 12 slow ALS or 6 fast ALS. Previous reports have shown the feasibility of this approach on neuronal cells using different dilutions ranging from 1% to 50% [70,71]. Cells were exposed to plasma for 12 h, to optimize leptin secretion [72]. The mature adipocytes were then treated with 1:100 diluted pooled plasma samples from either 12 healthy controls, 12 slow ALS, or 6 fast ALS (in differentiation maintenance medium). Cells were exposed to plasma for 12 h. When pre-treatment was conducted, adipocytes were treated with 10 μm of compound C (Sigma-Aldrich, St-Louis, MO, USA, #171261) or 1 μm of PP242 (Sigma-Aldrich, St-Louis, MO, USA, # 475988) for 1 h before addition of plasma samples. Cells were also treated with different concentrations of recombinant CCL16 (R&D systems, Minneapolis, MN, USA, # 802-HC-025) or recombinant sTNF-RII (MyBioSource, MBS343136, London, UK). After treatment, media was collected, and cells were harvested for future analysis.

#### Oil-Red O Coloration

The adipocytes were also cultured on a 10 mm coverslip to perform Oil-red O coloration and assure full maturity. Cells were fixed using 4% paraformaldehyde (PFA) for 30 min and washed four times with PBS. The cells were rinsed with isopropanol and stained with Oil Red O/ isopropanol solution for 15 min. Finally, cells were rinsed with isopropanol and distilled water.

### 4.6. ELISA

All ELISAs were performed as suggested by the manufacturer’s protocol. Leptin plasma levels were measured using a human leptin ELISA kit (Invitrogen, Waltham, MA, USA, #KAC2281) with samples diluted 1:100. Each patient’s samples were processed in duplicate. The same kit was used to measure leptin levels in cultures of human adipocytes’ undiluted supernatant. CCL16 plasma levels were measured using a CCL16 ELISA kit (Invitrogen, Waltham, MA, #EHCCL16) with 1:10 dilution. sTNF-RII plasma levels were measured using human sTNF-RII quantikine ELISA kit (R&D system, Minneapolis, MN, USA, #DRT200) with 1:10 dilution. Finally, a mouse leptin ELISA kit (Invitrogen, Waltham, MA, USA, #KMCC2281) was used to measure leptin levels in mouse adipocyte extracts (dilution 1:20). 

### 4.7. Statistics

We used student’s unpaired *t*-test for cytokines array in human and mouse plasma samples (Figure 1, Figure 2 and Figure 4) and for ELISA analysis in Figure 3 and Figure 5. Two-way ANOVA was used for analysis (Figure 6).

## Figures and Tables

**Table 1:** Demographics and disease characteristics of ALS patients and controls.

Characteristic	Age-Matched Controls (N = 38)	Sporadic ALS (N = 51)	*p*-Value
Age—yr.	65.86 ± 8.67	62.62 ± 10.62	0.1358
Female sex—no. (%) *	23 (60%)	20 (39.2%)	NA
Riluzole—no. (%)	NA	27 (53%)	NA
Weight—kg	73.80 ± 16.97	72.49 ± 16.84	0.7879
Weight loss—kg	NA	−4.53 ± 11.79	NA
BMI—kg/m^2^	26.83 ± 6.17	25.5 ± 6.13	0.3219
Disease duration—months	NA	33 ± 27.98	NA
ALSFRS-R—(/48)	NA	32.86 ± 9.29	NA
CVF—liter	NA	2.69 ± 0.98	NA
Fast progressor—no (%)	NA	11 (21.6%)	NA
Bulbar onset—no (%)	NA	11 (21.6%)	NA

Patients were recruited between July 2017 and December 2021 in two tertiary ALS centers in Canada. In the sALS group, 27 were taking riluzole but none were on edaravone. The mean disease duration was 33 months and patients had 33/48 on ALSFRS-R score. Eleven patients had bulbar onset. * Males were overrepresented in the ALS group. NA, not available.

**Table 2:** Clinical characteristics of slow and fast ALS.

Characteristic	Slow ALS (N = 40)	Fast ALS (N = 11)	*p*-Value
Rate of progression (ALSFRS-R points/12 weeks)	−1.683 ± 2.25	−7.884 ± 4.77	<0.0001
Age—yr.	62.75 ± 10.89	62.18 ± 10.02	0.877
Female—no. (%) *	18 (45%)	2 (18%)	NA
Weight—kg	74.62 ± 17.29	67.42 ± 15.03	0.161
Weight loss—kg	−2.456 ± 12.03	−10.03 ± 10.51	0.0703
BMI—kg/m^2^	26.18 ± 6.04	22.76 ± 6.53	0.1099
Disease duration—months	37.16 ± 30.40	16.07 ± 6.40	0.0276

* Males were overrepresented in the ALS group. *p*-Value NA for 2 Fast ALS female patients. NA, not available.

## Data Availability

The datasets used and/or analyzed during the current study are available from the corresponding author on reasonable request.

## Supplementary Materials

The following supporting information can be downloaded at: https://www.mdpi.com/article/10.3390/ijms24065065/s1.

## Abbreviations

ALS	Amyotrophic lateral sclerosis
Sals	Sporadic ALS
fALS	Familial ALS
ALSFRS-R	Revised ALS Functional Rating Scale
TDP-43	Transactive response (TAR) DNA binding protein 43
SOD1	Superoxide Dismutase one
UBQLN2	Ubiquiline-2
sTNF-RII	Soluble tumor necrosis factor – receptor II
AMPK	AMP-activated protein kinase
BMI	Body mass index
LIF	Leukemia inhibitory factor
TIMP-1	Tissue inhibitor of metalloproteinase 1
TIMP-2	Tissue inhibitor of metalloproteinase 2
SAA	Serum amyloid A
CCL1	C-C motif chemokine ligand 1
CCL2	C-C motif chemokine ligand 2
CCL4	C-C motif chemokine ligand 4
CCL16	C-C motif chemokine ligand 16
IFN-γ	Interferon-gamma
TNF-α	Tumor necrosis factor-alpha
mTOR	Mammalian target of rapamycin
PI3K/AKT	phosphoinositide-3-kinase–protein kinase B
