# Supplementary material for: Distinct Plasma Immune Profile in ALS Implicates sTNFR-II in pAMPK/Leptin Homeostasis
Source: Int J Mol Sci. 2023 Mar 7;24(6):5065. doi: 10.3390/ijms24065065 (PMC10049559; doi:10.3390/ijms24065065)
Supplement: Supplementary file 1 [file ijms-24-05065-s001.zip › ijms-2161410-supplementary.pdf]

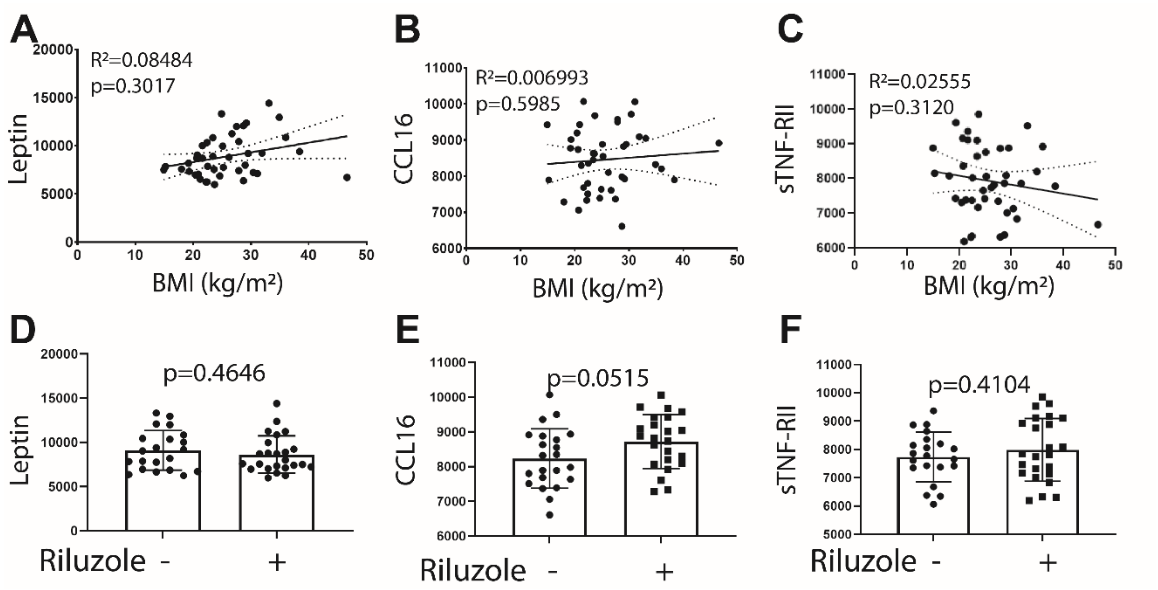

Supplementary fig. S1 a-c Correlation graph with simple linear regression for leptin vs BMI (a), CCL16 vs BMI (b) and sTNF-RII vs BMI (c). **d-f** No difference were observed in levels of leptin (d), CCL16 (e) and sTNF-RII (f) in patients treated with riluzole or not.

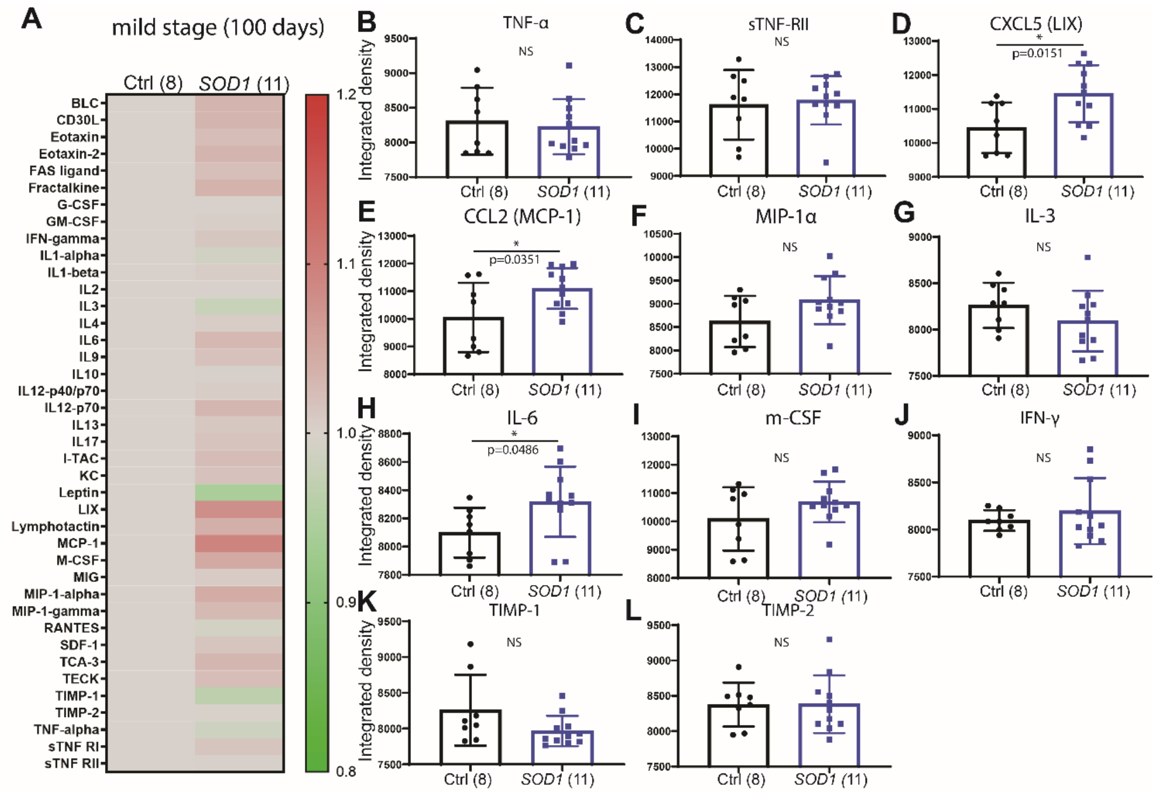

Supplementary fig. S2 Cytokines profile in mild stage SOD1^{G93A} mice. a Heat map illustrating cytokines changes (ratio) in SOD1^{G93A} mice as compared to non-transgenic age-matched mice. The number of mice is in parentheses. Significant changes were detected in LIX (**c**), MCP-1 (**e**) and Il-6 (**h**). Not significant (NS) changes were observed in (b, c, f, g, i, j, k, l) (*$p < 0.05$).

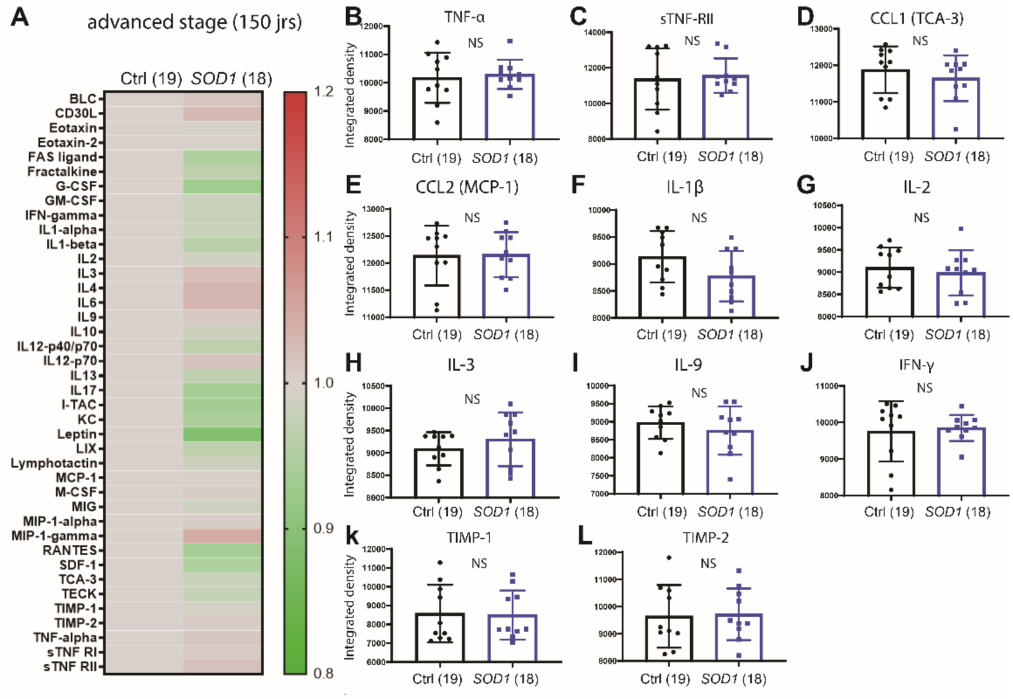

Supplementary fig. S3 Cytokines profile in advanced stage SOD1^{G93A} mice. a Heat map illustrating cytokines changes (ratio) in SOD1^{G93A} mice as compared to non-transgenic age-matched mice. The number of mice is in parentheses. No significant changes were detected in other cytokines. (b-l). NS, not siginficant.

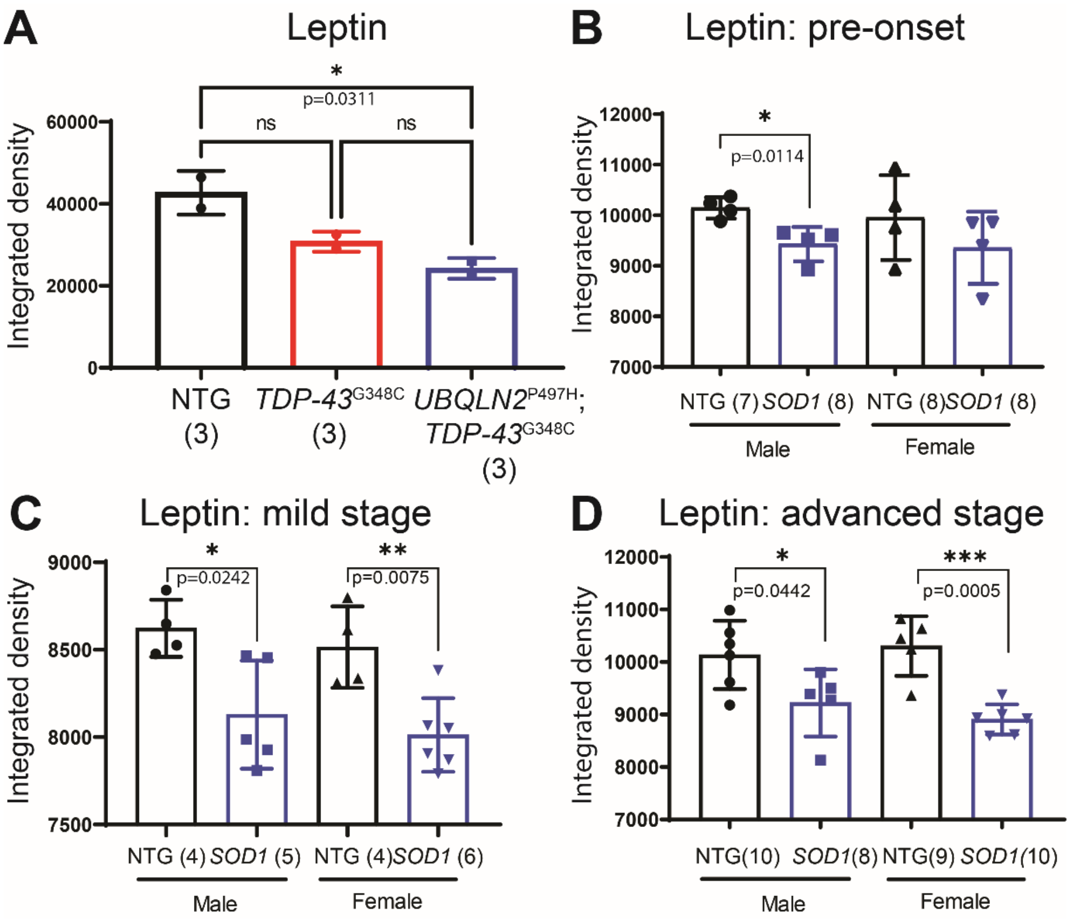

Supplementary fig. S4 a Leptin levels in plasma of non-transgenic (NTG), TDP-43^{G348C} mice and double transgenic UBQLN2^{P497H}; TDP-43^{G348C} at 8 months of age measured by cytokines array (one-way ANOVA). **b-d** Sex-dependent leptin levels measured by cytokines array in pre-onset (b), mild stage (c) and advanced stage (d) SOD1^{G93A} mice. (Data are mean±SEM *p≤0.05, **p<0.01, ***p<0.001, ns, not siginficant).
